# Struma Ovarii: A Thyroxine-Producing Ovarian Tumor in Pregnancy

**DOI:** 10.7759/cureus.18292

**Published:** 2021-09-26

**Authors:** Karim Botros, Nadiha Noor Chelsea, John Bermingham

**Affiliations:** 1 Obstetrics and Gynecology, University Hospital Waterford, Waterford, IRL; 2 Obstetrics and Gynecology, Royal College of Surgeons in Ireland, Dublin, IRL

**Keywords:** ovarian tumor, struma ovarii, pregnancy, dermoid cyst, benign

## Abstract

Struma ovarii is a rare dermoid tumor that consists of more than 50% thyroid tissue. The incidence of struma ovarii is reported to be 1% of all ovarian tumors and 2-5% of all ovarian teratomas. The authors present a case of struma ovarii diagnosed during the first trimester of pregnancy in a primigravida patient and discuss the clinical presentation, diagnosis, and management options for such rare tumors, both in and outside of pregnancy.

## Introduction

Struma ovarii is a rare ovarian tumor that can present with non-specific symptoms suggestive of a hyperthyroid state or perimenopausal symptoms in patients approaching menopause. A high level of clinical suspicion along with thorough clinical examination is crucial to diagnose such a tumor. Although benign forms are more common, malignant struma ovarii have also been documented, mainly as papillary thyroid carcinoma [1]. Struma ovarii frequently occurs as part of ovarian teratomas but can be identified with cystadenomas as well [2]. Surgical excision is the definitive form of treatment; however, the use of radioiodine therapy has also been indicated in recurrent or metastatic disease [3]. The authors report a case of a benign struma ovarii identified during pregnancy. 

## Case presentation

A 32-year-old primigravida patient of Irish-Caucasian descent visited the antenatal clinic for a booking visit on her 12th gestational week of a spontaneously achieved pregnancy. Ultrasound imaging showed the presence of a viable intrauterine pregnancy along with an incidental finding of a large 10.5 x 8.6 cm right adnexal cyst with multiple septations. The patient presented with no relevant past medical history. Her surgical history comprised of a previous laparoscopic right-sided ovarian cystectomy for a simple cyst diagnosed two years prior to her pregnancy. She had no known drug allergy and was not on any medication. She was a non-smoker with no prior alcohol use. She also had no relevant family history of malignancy.

Investigations

**Figure 1:**
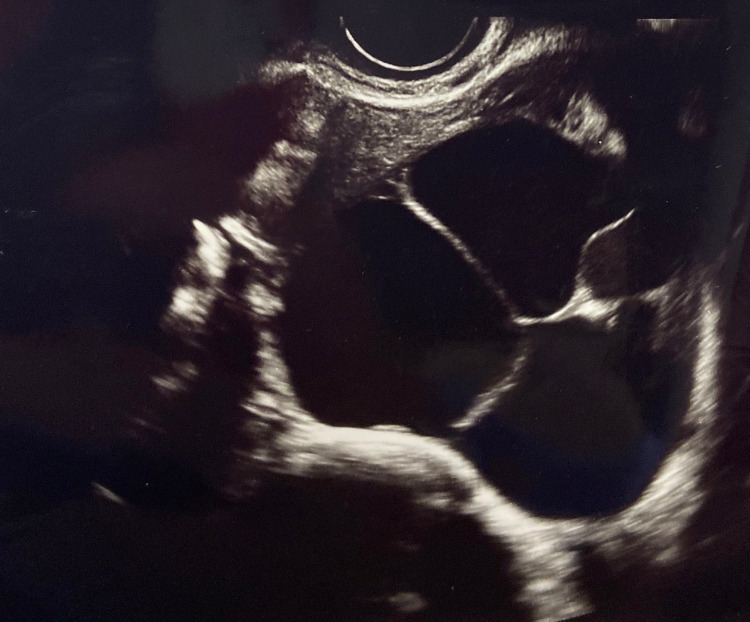
Right ovarian cyst with multiple septations

A repeat transvaginal ultrasound confirmed a large 10.5 x 8.6 cm right adnexal cyst with multiple septations as described previously (Figure 1).

Tumor markers such as alpha-fetoprotein (AFP), cancer antigen 125 (CA-125), and lactate dehydrogenase (LDH) were all within the normal range. 

Treatment

The patient was started on 400 mg vaginal progesterone once daily as a vaginal pessary at the time of diagnosis at 12 weeks of gestation. Progesterone was continued until 17 weeks of gestation when the patient underwent surgery as she developed lower abdominal pain. A unilateral right salpingo-oophorectomy was performed without any complications. The left ovary was normal intraoperatively. 

Outcome and follow-up

**Figure 2:**
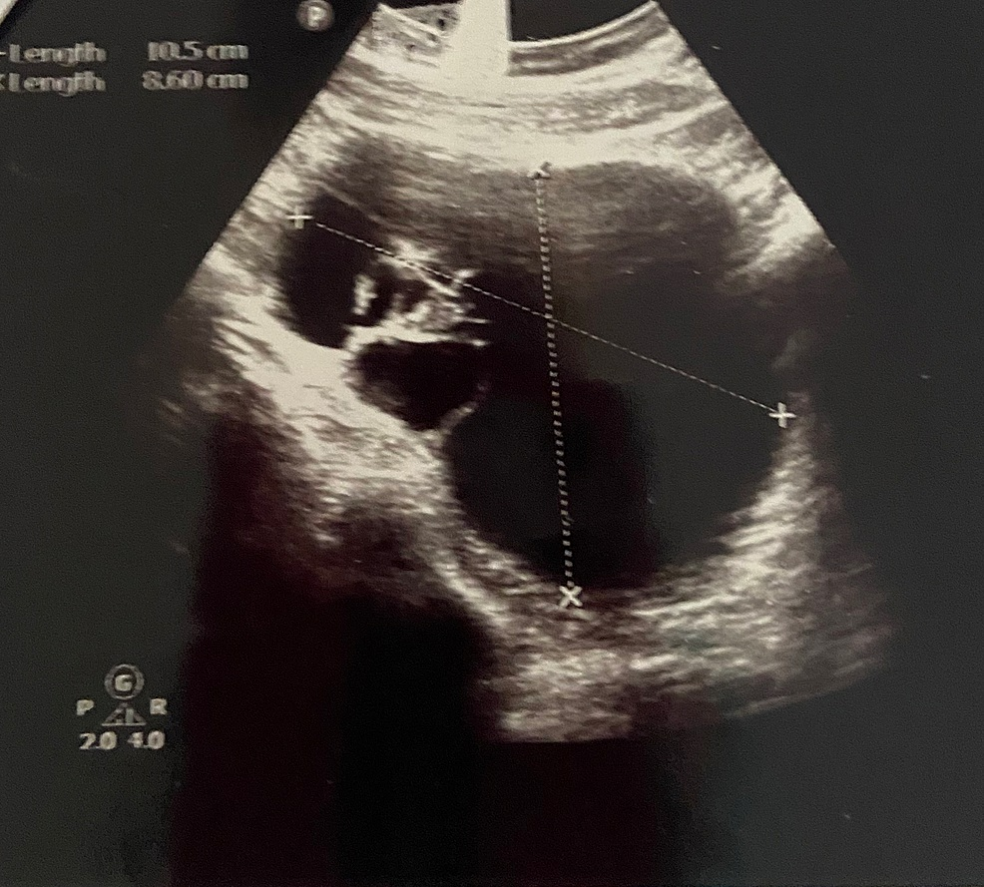
Transvaginal ultrasound revealing the right adnexal cyst

The patient was followed up in the antenatal clinic two weeks after her surgery. Her postoperative course was normal, and she recovered well. She delivered her baby at 40 weeks via an emergency cesarean section due to fetal distress. The transvaginal ultrasound followed by histological examination of the patient’s presenting right adnexal cyst reported a benign dermoid cyst, confirming the diagnosis of struma ovarii (Figure 2).

Her follow-up included a yearly ultrasound scan of the remaining left ovary along with observation of her thyroid-stimulating hormone (TSH), T3, and T4 levels. The patient's plan of care for the subsequent pregnancy will be close monitoring of her thyroid function tests every trimester as well as serial ultrasound scans to detect any possible recurrence on the other ovary.

## Discussion

The incidence of adnexal masses during pregnancy is approximately 0.2-2%, consisting mainly of benign tumors with dermoid cysts being the most common type [4-6]. Struma ovarii is a rare type of ovarian dermoid cyst [3,4].The incidence of struma ovarii is reported to be 1% of all ovarian tumors and 2-5% of all ovarian teratomas [2]. Studies have shown that thyroid tissue is common in dermoid cysts [3]. However, it represents only 5-15% of the overall structure while comprising over 50% in struma ovarii [3]. Yoo et al. reviewed 25 cases of struma ovarii over a period of 13 years to evaluate clinical features of the tumor [3]. The mean age of patients in their study was 45.3 years. The study found that 64% of the patients presented with symptoms of abdominal pain, abdominal mass, and vaginal bleeding, 24% had elevated CA-125 levels, and 16% had malignant struma ovarii. Furthermore, there were no cases of recurrent disease after patients underwent treatment [3]. On the other hand, Wee et al. reviewed 68 cases of struma ovarii with the objective of determining the ideal management and follow-up plans for such tumors. They concluded that simple laparoscopic surgery is the recommended treatment for struma ovarii because it leads to faster recovery and lower morbidity. The study found postoperative complications in only 10.3% of the cases. However, they showed that long-term postoperative follow-ups or investigations are not necessary for most patients [7].

Thus, struma ovarii usually presents as a rare and benign ovarian mass detected as an incidental finding in pregnancy, as in this case. However, histological diagnosis is required to rule out malignant forms. 

## Conclusions

In summary, a thorough history and clinical examination along with high clinical suspicion are key to recognize rare tumors, such as struma ovarii, especially in patients with symptoms of thyrotoxicosis along with a pelvic mass. While transvaginal ultrasound is the first modality of choice for investigation, diagnosis is only made on histology. The mainstay treatment for struma ovarii is suggested to be laparoscopic oophorectomy. However, ovarian cystectomy can be performed in premenopausal women with a follow-up ultrasound scan to check for recurrence. 

## References

[1] Zhang X, Axiotis C (2010). Thyroid-type carcinoma of struma ovarii. Arch Pathol Lab Med.

[2] Utsunomiya D, Shiraishi S, Kawanaka K (2003). Struma ovarii coexisting with mucinous cystadenoma detected by radioactive iodine. Clin Nucl Med.

[3] Yoo SC, Chang KH, Lyu MO, Chang SJ, Ryu HS, Kim HS (2008). Clinical characteristics of struma ovarii. J Gynecol Oncol.

[4] Sifakis S, Panayiotides IG, Angelakis E, Martavatzis N, Koumantakis E (2003). Benign struma ovarii complicating pregnancy: a case report and review of the literature. Arch Gynecol Obstet.

[5] de Haan J, Verheecke M, Amant F (2015). Management of ovarian cysts and cancer in pregnancy. Facts Views Vis Obgyn.

[6] Aggarwal P, Kehoe S (2011). Ovarian tumours in pregnancy: a literature review. Eur J Obstet Gynecol Reprod Biol.

[7] Wee JY, Li X, Chern BS, Chua IS (2015). Struma ovarii: management and follow-up of a rare ovarian tumour. Singapore Med J.

